# Long Non-Coding RNA and mRNA Expression Analysis in Liver of Mice With *Clonorchis sinensis* Infection

**DOI:** 10.3389/fcimb.2021.754224

**Published:** 2022-01-19

**Authors:** Su Han, Xue-Li Zhang, Xu Jiang, Xiang Li, Jian Ding, Li-Jiao Zuo, Shan-Shan Duan, Rui Chen, Bei-Bei Sun, Xin-Yi Hu, Yan-Nan Gao, Xiao-Li Zhang

**Affiliations:** ^1^ Department of Parasitology, Harbin Medical University, Harbin, China; ^2^ Department of Orthopaedic Surgery, The Fourth Affiliated Hospital of Harbin Medical University, Harbin, China; ^3^ Clinical Laboratory, Zhuhai Maternal and Child Health Hospital, Zhuhai, China; ^4^ Department of Stomatology, Laixi People’s Hospital, Qingdao, China

**Keywords:** clonorchiasis, liver, long non-coding RNAs, mRNA, microarray analysis

## Abstract

Clonorchiasis is recognized as an important zoonotic parasitic disease worldwide. However, the roles of host long non-coding RNAs (lncRNAs) and messenger RNAs (mRNAs) in the response to *Clonorchis sinensis* (*C. sinensis*) infection remain unknown. Here we compared the expression of lncRNAs and mRNAs in the liver tissue of mice infected with *C. sinensis*, in order to further understand the molecular mechanisms of clonorchiasis. A total of 388 lncRNAs and 1,172 mRNAs were found to be differentially expressed with absolute value of fold change (FC) ≥ 2.0 and *p* < 0.05 by microarray. Compared with controls, *Gm6135* and *4930581F22Rik* were the most over- and under-expressed lncRNAs; flavin-containing monooxygenase 3 (*Fmo3*) and deleted in malignant brain tumors 1 (*Dmbt1*) were the most over- and under-expressed mRNAs. Moreover, functional annotation showed that the significantly different mRNAs were related with “FOXO signaling pathway”, “Wnt signaling pathway”, and “AMPK signaling pathway”. Remarkably, lncRNA *Gm8801* were significantly correlated with mRNA glycerol-3-phosphate acyltransferase mitochondrial (*Gpam*), insulin receptor substrate 2 (*Irs2*), and tumor necrosis factor receptor superfamily member 19 (*Tnfrsf19*) in ceRNA networks. These results showed that the expression profiles of lncRNAs and mRNAs in the liver changed after *C. sinensis* infection. Our results provided valuable insights into the lncRNAs and mRNAs involved in clonorchiasis pathogenesis, which may be useful for future control strategies.

## Introduction

Clonorchiasis, caused by *Clonorchis sinensis* (*C. sinensis*), is an emerging zoonotic parasitic disease worldwide, distributed mainly in China, Korea, and northern Vietnam (Han et al., 2012; Lai et al., 2016). *C. sinensis*, belonging to family Opisthorchiidae (Ovchinnikov et al., 2015), has been classified by the International Agency for Research on Cancer (IARC) as a group 1 biocarcinogen in humans in 2009 (Bouvard et al., 2009). Humans become infected by eating freshwater fish with *C. sinensis* metacercariae. Metacercariae exist in the small intestine and then migrate *via* the ampulla of Vater to the biliary ducts (Lun et al., 2005). The adult fluke inhabits the biliary passages of humans and mammals and could cause epithelial hyperplasia of the biliary mucosa and even periductal fibrosis (Chung et al., 2004). Clinical manifestations of clonorchiasis vary from asymptomatic infections to severe morbidity and mortality, including epithelial hyperplasia, cholecystitis, periductal fibrosis, hepatic fibrosis, and cholangiocarcinoma (Keiser and Utzinger, 2005). Previously, we found that hepatic apoptosis and iron overload were involved in clonorchiasis (Han et al., 2017). In addition, an aberrant expression of hepatic microRNA has been associated with clonorchiasis (Han et al., 2016). However, the mechanism of liver/biliary injury, and whether other regulatory mechanisms or other small molecules are involved, remains unclear. Therefore, exploring the molecular mechanisms underlying clonorchiasis may contribute to the development of prevention measures and targeted drugs.

The majority of long non-coding RNAs (lncRNAs) are transcribed by RNA polymerase II as the product of alternative cleavage and splicing, with sizes greater than 200 nt in length (Quinn and Chang, 2016; Yamamura et al., 2018). LncRNAs are broadly classified into those that regulate gene expression in cis and in trans (Kopp and Mendell, 2018). Recently, lncRNAs are identified to bind with proteins, RNA, DNA, or their combination to play their functions (Fasolo et al., 2019; Hu et al., 2019). Acting as miRNA sponge, decoys, scaffolds, guides, and posttranslation regulators, lncRNAs could participate in various biological processes (Rinn and Chang, 2012; Mumtaz et al., 2017; Fernandes et al., 2019). Accumulating studies showed that lncRNAs were involved in inflammation, cancer, and autoimmunity (Fitzgerald and Caffrey, 2014; Marques-Rocha et al., 2015; Renganathan and Felley-Bosco, 2017).

Recent studies found that parasite infection changed the expression profiles of lncRNAs in the host. For example, numerous lncRNAs were differentially expressed in *S. japonicum*-infected liver and spleen compared to control (Xia et al., 2020). Furthermore, differentially expressed lncRNAs and their predicted target mRNAs were found associated with immunology and liver pathology (Xia et al., 2020). Besides, lncRNA WNT5b and H19 were found downregulated to regulate the metabolic reprogramming and differentiation of adipocytes in the protoscoleces of *Echinococcus granulosus*-infected mice (Lu et al., 2020). LncRNAs were also identified as important regulatory factors in regulating immunological stress during cystic echinococcosis (Yu et al., 2018). However, knowledge of lncRNAs and their biological functions in clonorchiasis remains limited.

This study used microarray analysis to assess the expression of lncRNAs and mRNAs in the liver tissue of mice infected with *C. sinensis*. Next, we analyzed the possible biological processes and pathways associated with *C. sinensis* infections. Our findings support this powerful new approach for the elucidation of the molecular mechanisms of clonorchiasis.

## Methods

### Mice, Parasites, and Infection

*C. sinensis* metacercariae were harvested from the cyprinid *Pseudorasbora parva* captured in Songhuajiang River, an endemic area of *C. sinensis* infection in China. Metacercariae were collected as described previously (Han et al., 2017). In brief, the fish were digested with a pepsin-HCl (0.6%) artificial gastric juice at 37°C for 12 h with vigorous shaking. With mesh sizes of 1,000 μm, 300 μm, and 106 μm, the digested mixture was collected and added to the conical-shaped glass and kept standing for 30 min. After discarding the supernatant, the precipitate was transferred to a glass dish and then the metacercariae were collected using a capillary pipette under a dissection microscope.

Male BALB/c mice (aged 8 weeks, 20 g) were obtained from the Harbin Medical University Laboratory Animal Center (Harbin, China). All the animals were housed in an air-conditioned room at 24°C under a 12-h dark/light cycle with free access to standard laboratory food and water. Mice were divided into two groups randomly with three mice per group, and each mice in the infected group was orally infected with 100 metacercariae, while the control group was given the same volume of 0.9% NaCl. The detection of eggs in feces indicates the successful establishment of an animal model of *C. sinensis* infection (Hong et al., 2003). Thus, feces were collected every day and microscopically examined by the Kato–Katz method (Hong et al., 2003).

*C. sinensis* undergoes rapid development, and the adults develop matured at 4 week postinfection (wpi) after being infected by metacercariae (Lun et al., 2005). Our previous study also found hepatocyte apoptosis and iron overload in *C. sinensis*-infected rats at 4 wpi (Zhang et al., 2008; Han et al., 2017). Therefore, the mice in both infected and control groups were sacrificed under ether anesthesia by cervical dislocation at 4 wpi. Then, livers were removed and immediately stored at -80°C after being washed by nuclease-free PBS. All animal care and experimental procedures were conducted according to the guidelines for animal use in toxicology. The study protocol was approved by the Animal Care and Use Committee of the Harbin Medical University.

### RNA Extraction

Total RNA was extracted from 100 mg of tissue using TRIzol Reagent (Life Technologies, Carlsbad, CA, USA) following the manufacturer’s instructions. RNA quantity was measured using a SmartSpec Plus spectrophotometer (Bio-Rad, Hercules, CA, USA). RNA purity was evaluated by the A260/A280 ratio. Total RNA was quantified using a NanoDrop ND-2000 (Thermo Scientific, Waltham, MA, USA), and RNA integrity was assessed using an Agilent Bioanalyzer 2100 (Agilent Technologies, Santa Clara, CA, USA).

### Microarray Analysis

Three pairs of liver samples were selected for lncRNA microarray analysis using an Agilent Mouse Gene Expression Array (8*60K, design ID: 028005). The microarray contains 55,681 probes for 39,430 mouse mRNA and 16,251 mouse lncRNAs, which are derived from RefSeq Build 37, Ensemble Release 55, Unigene Build 176, GenBank (Apr 2009), and RIKEN 3. Sample preparation, microarray hybridization, and washing were performed based on the manufacturer’s standard protocols with minor modifications. After that, the arrays were scanned using an Agilent Scanner G2505C microarray scanner (Agilent Technologies). Raw data were extracted using Feature Extraction (version 10.7.1.1; Agilent Technologies). The microarray profiling was conducted in the laboratory of the OE Biotechnology Company in Shanghai, People’s Republic of China.

### Bioinformatics Analysis

Firstly, quantile normalization and subsequent data processing were done through GeneSpring software (version 12.0; Agilent Technologies). Differentially expressed genes (mRNAs and lncRNAs) were identified by the absolute value of (FC) > 2 and *p* value < 0.05 (Student’s t test). Gene ontology (GO) analysis was used to identify the potential biological functions of the differentially expressed mRNAs (www.geneontology.org). Biological process (BP), cellular component (CC), and molecular function (MF) were involved in the GO terms. Fisher’s exact test was applied to classify the GO categories. Enrichment scores were calculated based on log10 (*p*-value). The lower the *p*-value, the more significant the GO term (*p* < 0.05 is required). Furthermore, the Kyoto Encyclopedia of Genes and Genomes (KEGG) pathway analysis (http://www.kegg.jp) was applied to predict the possible pathways. Hierarchical clustering was used to display the distinguishable genes’ expression pattern among six liver tissue samples (Cluster 3.0 and TreeView 2.0).

In order to further predict the potential regulatory relationship between mRNA and lncRNA, a ceRNA network was constructed by combining our previous miRNA expression profiling date (**Additional File: Table S1**). The processing of construction was as follows: i) Pearson’s correlation coefficients were used for analyzing lncRNAs and their associated mRNAs, based on correlation coefficients ≥0.90 and *p* < 0.01 considered statistically significant (**Additional File: Table S4**). ii) mRNA and lncRNA shared at least 4 miRNAs. iii) *p* ≤ 0.05. Cytoscape software 3.1.1 (US National Institute of General Medical Sciences) was used for constructing the ceRNA network.

### Quantitative Reverse Transcription-Polymerase Chain Reaction

Total RNA was extracted from the liver, and the integrity was evaluated using agarose gel electrophoresis stained with ethidium bromide. Quantitative reverse transcription-polymerase chain reaction (qRT-PCR) was performed on six differentially expressed mRNAs and five lncRNAs to confirm the results of the microarray analysis. Based on the protocols provided by the manufacturers, a SYBR Green RT-PCR Kit (TransStart Top Green qPCR SuperMix, TransGen Biotech Co., Ltd., Beijing, China) on a Light Cycler 96 system (Roche, Indianapolis, Indiana) was used for qRT-PCR. Primers for the lncRNAs and mRNAs are shown in **Table 1**. Threshold cycle (Ct) values were used to quantify the expression levels of lncRNAs and mRNAs. Glyceraldehyde 3-phosphate dehydrogenase (GAPDH) was used as an internal control for measurement of lncRNAs and mRNAs using the 2-ΔΔCt method. Three biological replicates were conducted for each sample.

**Table 1:** Primers used in qPCR detection of selected mRNAs and lncRNAs.

Gene name		Forward primer	Reverse primer
mRNAs	*Pax2*	ACGAGACTGGCAGCATCAA	CGGGTTCTGTCGCTTGTATT
	*Btg2*	ATGAGCCACGGGAAGGGAA	TTGGACGGCTTTTCGGGAA
	*Dmbt1*	CCCTGGACGATGTAGAGTGC	GGACGGGTGATGTTGAGAAA
	*Irs2*	ACCGACTTGGTCAGCGAAG	CACGAGCCCGTAGTTGTCAT
	*Tnfrsf19*	ATGGCTCTGAGCTGTCATGC	GCCAGGCATCAGAAAACTCCG
	*Gpam*	CCGCTCGAGGGCCTTGTGAGCAGAAAATC	ATTTGCGGCCGCTACAAATGCAAGCTCCTTGG
	*GAPDH*	GAGAGACCCTCACTGCTG	GATGGTACATGACAAGGTGC
lncRNAs	*Fate1*	GATGGAGCTATCCCCTCCTC	CCTAGCCTTCCAGCAGCTAA
	*Meg3*	CTGCCCATCTACACCTCACG	CTCTCCGCCGTCTGCGCTAGGGGCT
	*Snhg3*	TTCAAGCGATTCTCGTGCC	AAGATTGTCAAACCCTCCCTGT
	*Gm6135*	CATGGGATGTGAGCAGTCTT	TGAGGATTCAGGCTGGAGTG
	*Gm8801*	CCCCAAACCCTTTCCAGTAT	GTGCAGTGCAGTGGGATAGA

Pax2, paired box 2; Btg2, B cell translocation gene 2, anti-proliferative; Dmbt1, deleted in malignant brain tumors 1; Irs2, insulin receptor substrate 2; Tnfrsf19, tumor necrosis factor receptor superfamily, member 19; Gpam, glycerol-3-phosphate acyltransferase, mitochondrial; Fate1, fetal and adult testis expressed 1; Meg3, maternally expressed 3; Snhg3, small nucleolar RNA host gene 3; Gm6135, prediticted gene 6135; Gm8801, protein phosphatase 1, regulatory subunit 10 pseudogene; GAPDH, Glyceraldehyde 3-phosphate dehydrogenase.

### Statistical Analysis

PRISM version 8.0 software (GraphPad, San Diego, CA, USA) was used for data analysis. All data are expressed as mean ± standard deviation (SD). Student’s t-tests were employed to analyze qRT-PCR data. *p* < 0.05 was considered statistically significant.

## Results

### Differentially Expressed lncRNAs and mRNAs

**Figure 1:**
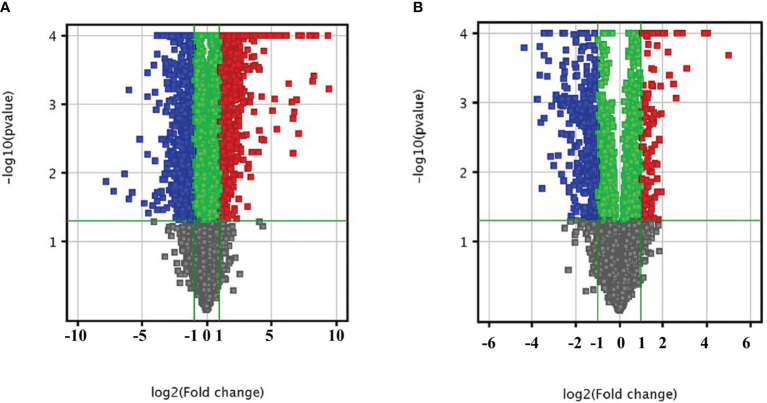
Bioinformatics analysis of differentially expressed mRNAs **(A)** and lncRNAs **(B)** in liver tissue infected with *C. sinensis*. The vertical axis corresponds to the negative logarithm of p value with base 10 (-log10(p value)), and the horizontal axis represents the logarithm of fold change with base 2 (log2(fold change)). The significantly up- and downregulated RNAs are presented as red or blue squares, respectively.

**Figure 2:**
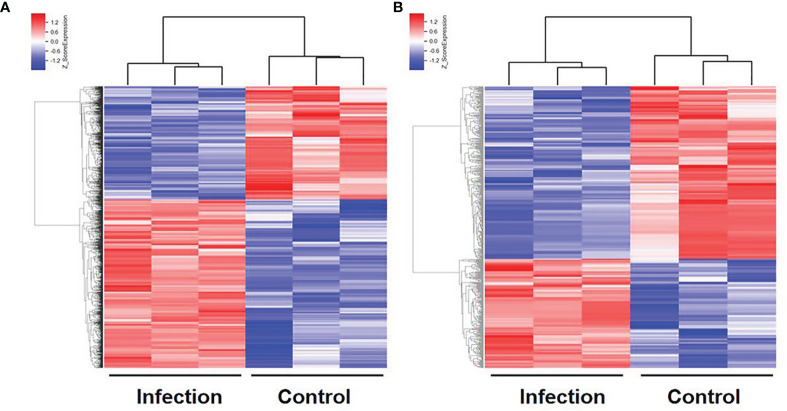
Hierarchical clustering of differentially expressed mRNAs **(A)** and lncRNAs **(B)** in the liver with *C. sinensis* infection. Blue indicates decreased relative expression, and red indicates increased relative expression.

In order to explore the lncRNA and mRNA expression profiles in the liver in response to *C. sinensis* infection, microarray analysis was employed to compare expressions in uninfected and *C. sinensis*-infected liver tissues. A total of 388 lncRNAs (115 up and 273 down, **Additional File: Table S2**) and 1,172 mRNAs (687 up and 485 down, **Additional File: Table S3**) were found to be differentially expressed with absolute value of FC ≥ 2.0 and *p* < 0.05 by microarray. The differences in lncRNA and mRNA expression profiles between the two groups are shown in **Figure 1**. Compared with the control group, *4930581F22Rik* (absolute value of FC = 140.37) was the most downregulated lncRNA, and *Gm6135* (absolute value of FC = 44.27) was the most upregulated lncRNA in the infected group. Furthermore, *Dmbt1* (absolute value of FC = 294.36) was the most downregulated mRNA, encoding DMBT1 protein, whereas *Fmo3* (absolute value of FC = 703.50) was the most upregulated mRNA, encoding FMO3 protein. In order to further understand the similarities at the transcriptomic level, cluster analysis was performed on all the differentially expressed genes. The infected group and the control group clustered separately in the heat map (**Figure 2**). These results indicated that altered expressions of lncRNAs and mRNAs may play an important role in clonorchiasis.

### The qRT-PCR Validation

**Figure 3:**
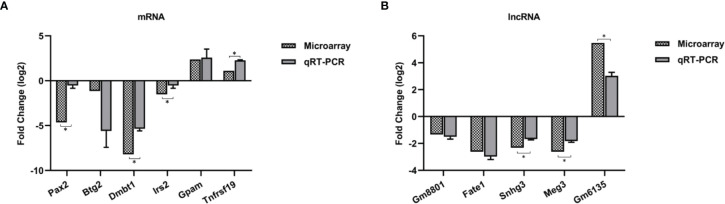
Validation for the expression of 6 differentially expressed mRNAs **(A)** and 5 lncRNAs **(B)** by qRT-PCR. Three biological repeats were included in each gene. *p < 0.05.

To evaluate the reliability of microarray results, six mRNAs (*Pax2*, *Btg2*, *Dmbt1*, *Irs2*, *Tnfrsf19*, and *Gpam*) and five lncRNAs (*Fate1*, *Meg3*, *Snhg3*, *Gm6135*, and *Gm8801*) were selected for qRT-PCR. *Dmbt1* was the most downregulated mRNA, whereas *GM6135* was the most upregulated lncRNA. The lncRNA *Gm8801* was chosen because it was strongly correlated with mRNA *Gapm*, *Irs2*, and *Tnfrsf19* in our ceRNA network. Beside, two mRNAs (*Pax2* and *Btg2*) and three lncRNAs (*Fate1*, *Meg3*, and *Snhg3*) were randomly chosen. The qRT-PCR results were consistent with the results of the microarray (**Figure 3**).

### GO and KEGG Enrichment Analyses

**Figure 4:**
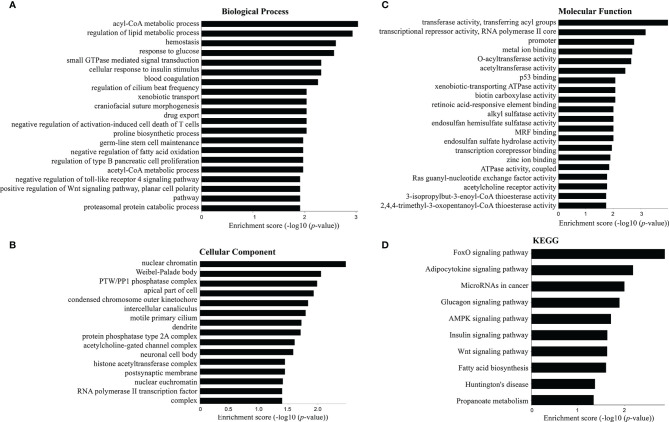
Top 20 terms in the gene enrichment and pathway analysis of differentially expressed mRNAs induced by *C. sinensis* infection. **(A)** Biological process. **(B)** Cellular component. **(C)** Molecular function. **(D)** The most significant KEGG pathway for the differentially expressed mRNAs.

GO analysis and KEGG enrichment analysis were applied to assess the function roles of differentially expressed mRNAs. **Figure 4** shows the top 20 terms in the gene enrichment and pathway analyses of differentially expressed mRNAs induced by *C. sinensis* infection. For instance, differentially expressed mRNAs were mainly involved in “response to glucose”, “regulation of lipid metabolic process”, “negative regulation of fatty acid oxidation”, “negative regulation of toll-like receptor 4 (TLR4) signaling pathway”, and “positive regulation of Wnt signaling pathway, planar cell polarity pathway” (**Figure 4A**). The cellular components included “protein phosphatase type 2A complex” and “RNA polymerase II transcription factor complex”, while molecular functions were associated with “biotin carboxylase activity” and “ATPase activity, coupled”. KEGG pathway enrichment analysis was used to further understand the predicted biological functions of the mRNAs. The enriched pathways included “FoxO signaling pathway”, “AMPK signaling pathway”, “Wnt signaling pathway”, “Glucagon signaling pathway”, and “Insulin signaling pathway”. These results showed the disorder of lipid metabolism and glucose metabolism in clonorchiasis.

### Co-Expression Networks of lncRNAs and mRNAs

**Figure 5:**
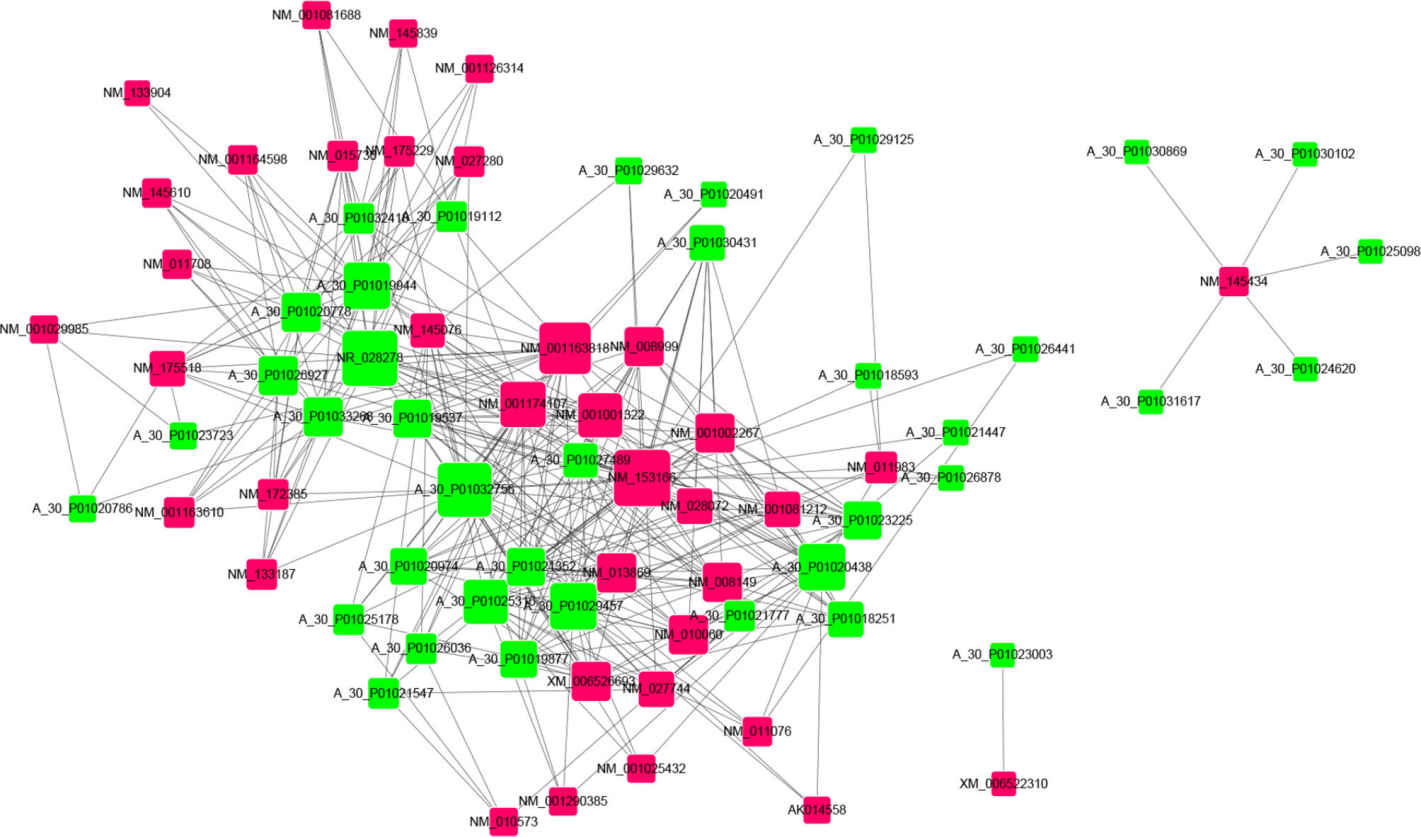
CeRNA network of the differentially expressed lncRNAs with their associate mRNAs. Co-expression lncRNA–mRNA pairs were identified using strict screening criteria (correlation coefficient ≥ 0.90 and p ≤ 0.01). Different colors were used to show different genes, with green for lncRNAs and red for mRNAs.

**Figure 6:**
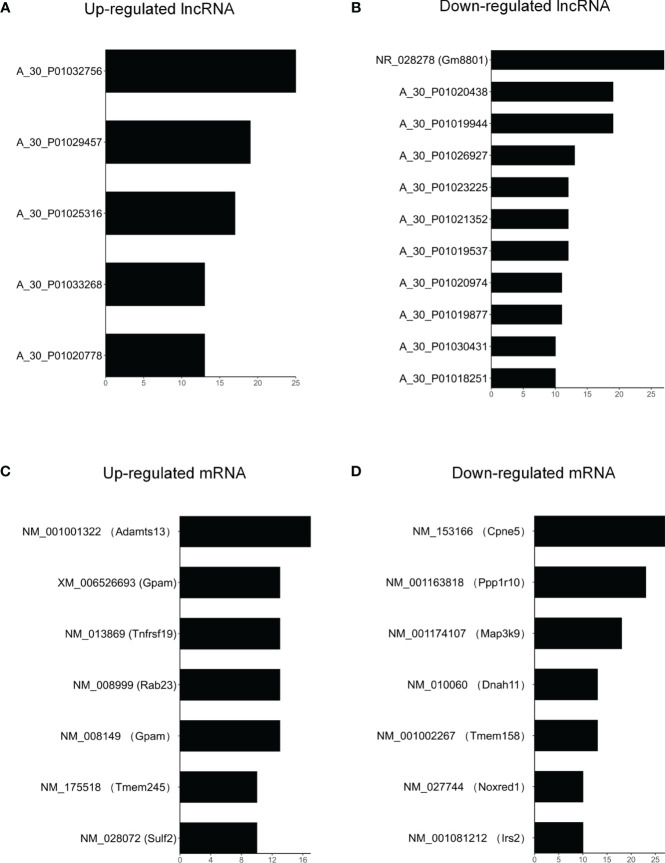
The top correlated lncRNA-mRNA pairs. **(A)** Up-regulated and **(B)** down-regulated lncRNAs with correlation degrees > 10. **(C)** Up-regulated and **(D)** down-regulated mRNAs with correlation degrees > 10. Correlation levels of 38 lncRNAs and 37 mRNAs were evaluated by correlation degrees in the network. The degree is equal to counts of significantly correlated lncRNA-mRNA pairs (P < 0.01) involving the corresponding lncRNA or mRNA.

In order to analyze correlations among the differentially expressed lncRNAs and mRNAs and reveal their underlying mechanisms, a ceRNA was constructed (**Figure 5** and **Table S5**). In these networks, each lncRNA can be related to one or more mRNAs, and each mRNA can also be associated with one or more lncRNAs. For example, mRNA *Gpam* was upregulated and associated with *GM8801* (*A_55_P2114498*), *A_30_P01020438*, *A_30_P01029457*, and 10 other lncRNAs. Both mRNA *Irs2* (downregulated) and *Tnfrsf19* (upregulated) were found sharing 13 lncRNAs including *Gm8801*, *A_30_P01020438*, and *A_30_P01029457*. in the network. Additionally, the scatter plots with correlation of selected lncRNA *Gm8801* and mRNA *Gpam*, *Tnfrsf19*, and *Irs2* in the co-expression network are shown in **Figure S1**, respectively. LncRNA *Gm8801* was found negatively correlated with mRNA *Gpam* (*R^2^ = 0.9301*) and *Tnfrsf19* (*R^2^ = 0.8745*), while it was positively correlated with mRNA *Irs2* (*R^2^ = 0.8252*), respectively. The lncRNAs and mRNAs with the highest correlation degrees are listed in **Figure 6**, respectively. Among the upregulated lncRNAs, *A_50_P01032756* was the one with the highest mRNA correlation degree, while lncRNA *Gm8801* was the top one regarding the mRNA correlation degree. As for the mRNAs, up-regulated Adamts13 was correlated with the most lncRNAs, while and down-regulated Cpne5 was the top one regarding the lncRNAs correlation degree.

### Verification of the Correlation Between lncRNA Gm8801 and mRNAs

**Figure 7:**
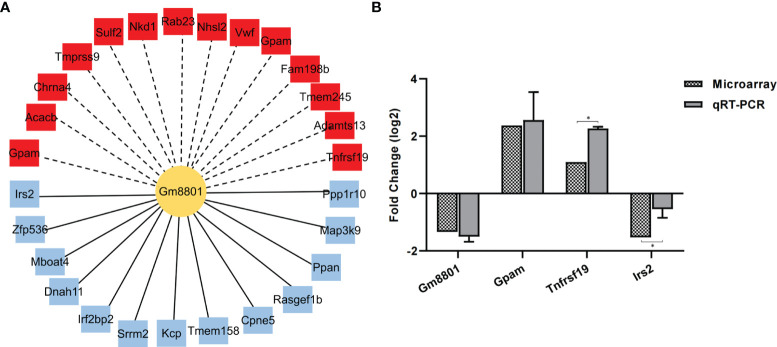
The correlation between mRNAs and lncRNA *Gm8801*. **(A)** The mRNAs correlated with *Gm 8801* lncRNA. The red color indicates upregulation, while the blue color indicates downregulation. The solid lines mean positive correlation, and the dashed line means negative correlation. **(B)** The levels of mRNA *Irs2*, *Gpam*, *Tnfrsf19*, and lncRNA *Gm8801*. *p < 0.05.

To verify the reliability of the correlation network, we selected lncRNA *Gm8801* (downregulated), which was the only annotated lncRNA in the network, as the central lncRNA to build a subnetwork of the lncRNA–mRNA correlation. In this subnetwork, 13 mRNAs positively correlated with lncRNA *Gm8801* expression were downregulated, while 14 mRNAs negatively correlated with *Gm8801* expression were upregulated (**Figure 7A**). Subsequently, among the mRNAs correlated with *Gm8801* lncRNA, mRNAs *Gpam*, *Tnfrsf19*, and *Irs2* were selected to be preliminarily verified by qPCR. As shown in **Figure 7B**, mRNA *Irs2* was decreased, while mRNA *Gpam* and *Tnfrsf19* were increased at the same time point coinciding with the decrease of lncRNA *Gm8801* in the *C. sinensis*-infected group. These results indicated that mRNA *Irs2* was positively correlated with lncRNA *Gm8801*, while mRNA *Gpam* and *Tnfrsf19* were negatively correlated with *Gm8801*, which is consistent with the prediction.

## Discussion

Previous studies on the interactions between parasites and their hosts have focused mainly on protein-coding genes (Melo et al., 2013; He et al., 2016). Recently, it was reported that lncRNAs play important roles in gene imprinting, cell differentiation, and tumorigenesis and are also involved in host–pathogen interactions (Jathar et al., 2017). For instance, RNA-Seq revealed that differentially expressed lncRNAs of *Schistosoma mansoni* were mainly involved in RNA-dependent DNA replication and G-protein coupled receptor (GPCR) signaling pathway/GPCR activity (Vasconcelos et al., 2017). However, the expression profiles of lncRNAs specific to *C. sinensis* infection were unclear. In this study, the expression profiles of lncRNAs and mRNAs in the liver of *C. sinensis*-infected mice at 4 wpi were fully explored using microarray analysis.

In this study, a total of 388 lncRNAs and 1,172 mRNAs were dysregulated in *C. sinensis*-infected group. As the most upregulated lncRNA, *Gm6135* is primarily localized to the cytoplasm and participates in posttranslational modification (Rashid et al., 2016; Ji et al., 2019). LncRNA *Gm6135* could increase the expression of toll-like receptor 4 (TLR4), by competitive binding to miR-203-3p, further activating the secretion of pro-inflammatory cytokines in mice (Ji et al., 2019; Loganathan et al., 2020). A previous study showed that TLR4 was upregulated in activated hematopoietic stem cells (HSCs) and myofibroblasts infected with *C. sinensis* (Yan et al., 2015). Thus, differentially expressed lncRNA *Gm6135* may regulate the expression of TLR4 to induce inflammatory response in clonorchiasis. However, the role of *4930581F22Rik* (the most downregulated lncRNA) has not been described clearly. The FMO3 protein encoded by *Fmo3* (the most upregulated mRNA) is found associated with gallstone formation in mice (Chen et al., 2019). FMO3 protein and its metabolite (TMAO) participate in the TAF–FMO3–TMAO pathway, which could regulate lipid metabolism (Schugar and Brown, 2015; Warrier et al., 2015), while the downregulated mRNA *Dmbt1* could encode the DMBT1 protein, which is related to liver injury and repair mechanisms in liver diseases (Bisgaard et al., 2002; Deng et al., 2012). Therefore, we speculate that mRNA *Fmo3* and *Dmbt1* might contribute to lipid metabolism and liver injury in clonorchiasis.

KEGG enrichment analysis showed that the top 20 predicted pathways mainly participated in “FOXO signaling pathway”, “Wnt signaling pathway”, and “AMPK signaling pathway”. For instance, mRNA *Irs2* and *Crebbp* were rich in “FOXO signaling pathway”, which was involved in cellular differentiation, apoptosis, cell proliferation, DNA damage and repair, and oxidative stress (Gomes et al., 2008; Vurusaner et al., 2012). *Nk1d1* and *Crebbp* mRNA were rich in “Wnt signaling pathway”, which played critical roles in precancerous lesions, malignant transformation of liver cells, and malignant expansion of cancer cells (Zhang et al., 2017). *Nr1d1* mRNA was also involved in “negative regulation of TLR4 signaling pathway” in biological processes. TLR contributes to host defense by regulating host innate and adaptive immune responses (Iwasaki and Medzhitov, 2004). TLR4 was involved in immune responses, including increased levels of tumor necrosis factor alpha and interferon gamma during clonorchiasis (Yan et al., 2015). Interestingly, we also found that *Irs2* and *Gpam* mRNA participated in “response to glucose” in biological processes. Moreover, “regulation of lipid metabolic process”, “negative regulation of fatty acid oxidation”, “Glucagon signaling pathway”, and “Fatty acid biosynthesis” demonstrated to be involved in *C. sinensis* infection. Therefore, we speculated that *C. sinensis* infection affected the cell function as well as lipid and glucose metabolism of the host.

Based on our previous miRNA expression profiling data (**Additional File: Table S1**) and differentially expressed lncRNAs and mRNAs, a ceRNA network was constructed, in order to explore the functions of lncRNAs. In ceRNA networks, mRNA *Gpam* and *Tnfrsf19* were negatively correlated with lncRNA *Gm8801* and *A_30_P01020438*, indicating an upregulation of *Gpam* and *Tnfrsf19* mRNA in the *C. sinensis*-infected group. *Gpam* mRNA is correlated with T lymphocyte proliferation, lipid metabolism, defense against viruses, and IL-2 secretion (Brockmoller et al., 2012; Warrier et al., 2015). In addition, *Tnfrsf19* mRNA belongs to the tumor necrosis factor receptor superfamily, which commonly transduces cytokine signals and is associated with poor prognosis in various types of cancer (Spanjaard et al., 2007; Fafilek et al., 2013; Loftus et al., 2013; Schon et al., 2014). Otherwise, *Irs2* mRNA was positively correlated with *Gm8801* and *A_30_P01020438* lncRNA, indicating a downregulation of *Irs2* in the *C. sinensis*-infected group. *Irs2* plays requisite roles in cell proliferation, apoptosis, migration, and tumor invasion (Lea et al., 2014; Zhao et al., 2014). Moreover, *Irs2* mRNA is also important in hepatic nutrient homeostasis, as it mediates the anabolic effects of insulin through the PI3K-Akt cascade (Rother et al., 1998; Andersson et al., 2004) and suppresses gluconeogenesis (Valverde et al., 2003). Therefore, we hypothesized that lncRNA *Gm8801* and *A_30_P01020438* might play important roles in host metabolism and liver injury in clonorchiasis by regulating its co-expressed mRNA.

In this study, a microarray approach was used to explore the differential expression profiles of lncRNAs and mRNAs in clonorchiasis for the first time. However, there were some limitations that should be addressed. We only analyzed the expression profiles of lncRNAs and mRNAs at 4 weeks after being infected with *C. sinensis*, which corresponds to the early stage. However, *C. sinensis* infection is a dynamic process; the expression profiles of lncRNAs and mRNAs are also needed for detection at the late stages of infection. Moreover, the potential interaction between LncRNA and mRNA is needed to validate in the future.

## Conclusions

In this study, we investigated the differential expression profiles of lncRNAs (388) and mRNAs (1,172) in the livers of mice infected with *C. sinensis*. Our results provided valuable insights into the lncRNAs and mRNAs involved in clonorchiasis, which may be useful for future control strategies. In the future, the functions of the differentially expressed lncRNAs and mRNAs in the pathogenesis of *C. sinensis* infection need to be further verified.

## Data Availability Statement

The datasets presented in this study can be found in online repositories. The names of the repository/repositories and accession number(s) can be found below: https://www.ncbi.nlm.nih.gov/geo/, accession ID: GSE188841.

## Ethics Statement

The animal study was reviewed and approved by the Care and Use of Laboratory Animals of the Ministry of Science and Technology, China.

## Author Contributions

SH and Xi-LZ offered advice for the article framework and conceived the conception and design of the trial. SH and Xi-LZ drafted the first manuscript and participated in trial communication and monitoring. Xu-LZ, XJ, XL, JD, JZ, SD, RC, BS, YH and NG modified the manuscript the carried out statistical calculations. All authors participated in revision of the manuscript and approved the final version.

## Funding

This work was supported by the National Natural Science Foundation of China (81971958, 81601785, and 81401684), Heilongjiang Provincial Natural Science Foundation of China (LH2020H016), Post-doctoral Science Foundation (LBH-Q174), and Postgraduate Research & Practice Innovation Program of Harbin Medical University (YJSKYCX2019-67HYD). The funder had no role in the study design, data collection and analysis, decision to publish, or preparation of the manuscript.

## Conflict of Interest

The authors declare that the research was conducted in the absence of any commercial or financial relationships that could be construed as a potential conflict of interest.

## Publisher’s Note

All claims expressed in this article are solely those of the authors and do not necessarily represent those of their affiliated organizations, or those of the publisher, the editors and the reviewers. Any product that may be evaluated in this article, or claim that may be made by its manufacturer, is not guaranteed or endorsed by the publisher.

## Supplementary Material

Supplementary Figure 1 The scatter plots with correlation of selected lncRNA and mRNA in co-expression network. LncRNA *Gm8801* relative expression levels and *Gpam* mRNA **(A)**, *Tnfrsf19* mRNA **(B)**, *Irs2* mRNA **(C)** relative expression levels.

Supplementary Table 1 The expression profile of differentially expressed miRNAs in the liver of control and infected groups.

Supplementary Table 2 The expression profile of differentially expressed lncRNAs in the liver of control and infected groups.

Supplementary Table 3 The expression profile of differentially expressed mRNAs in the liver of control and infected groups.

Supplementary Table 4 Raw data from the lncRNA-mRNA correlation analysis.

Supplementary Table 5 Raw data from ceRNA network analysis.

## Abbreviations

GO, Gene Ontology; KEGG, Kyoto Encyclopedia of Genes and Genomes; lncRNA, long non-coding RNA; mRNA, messenger RNA; qPCR, real-time quantitative PCR; TLR4, toll-like receptor; *Pax2*, paired box 2; *Btg2*, B cell translocation gene 2, anti-proliferative; *Dmbt1*, deleted in malignant brain tumors 1; *Irs2*, insulin receptor substrate 2; *Tnfrsf19*, tumor necrosis factor receptor superfamily, member 19; *Gpam*, glycerol-3-phosphate acyltransferase, mitochondrial; *Fate1*, fetal and adult testis expressed 1; *Meg3*, maternally expressed 3; *Snhg3*, small nucleolar RNA host gene 3; *Gm6135*, prediticted gene 6135; *Gm8801*, protein phosphatase 1, regulatory subunit 10 pseudogene; *Crebbp*, CREB (cyclic adenosine monophosphate (cAMP) response element-binding protein; GAPDH, glyceraldehyde 3-phosphate dehydrogenase.
