# Supplementary figures and images for: Long Non-Coding RNA and mRNA Expression Analysis in Liver of Mice With Clonorchis sinensis Infection
Source: Front Cell Infect Microbiol. 2022 Jan 19;11:754224. doi: 10.3389/fcimb.2021.754224 (PMC8807509; doi:10.3389/fcimb.2021.754224)

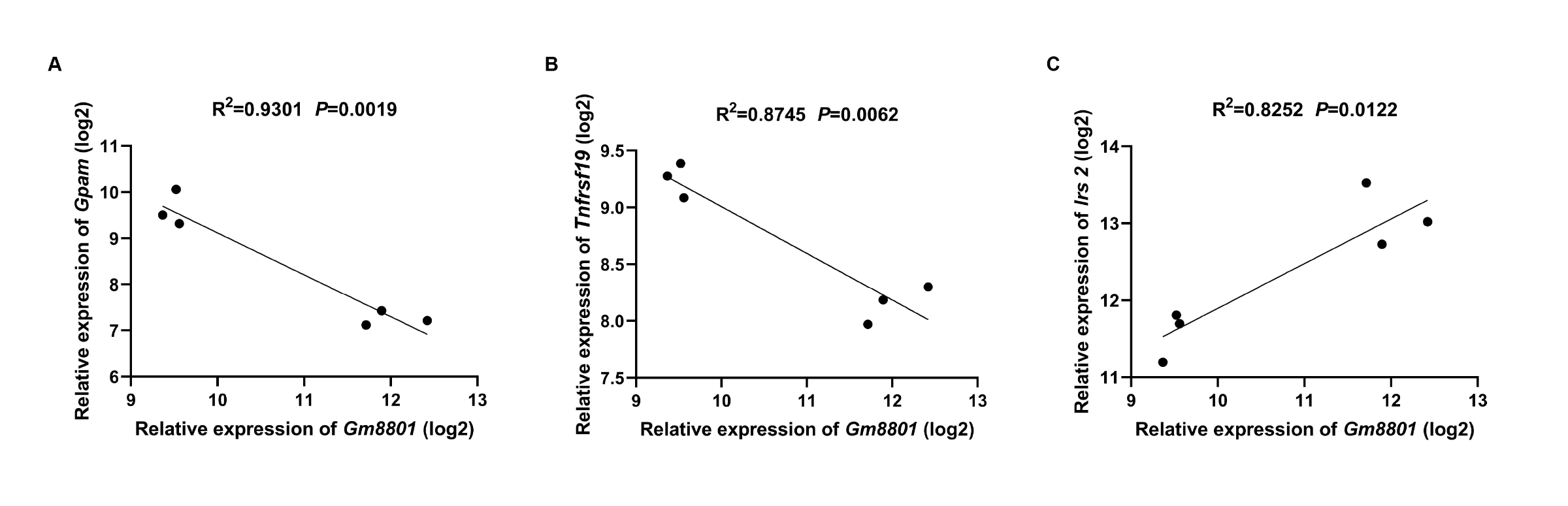

Supplement: Supplementary Figure 1 — The scatter plots with correlation of selected lncRNA and mRNA in co-expression network. LncRNA Gm8801 relative expression levels and Gpam mRNA (A), Tnfrsf19 mRNA (B), Irs2 mRNA (C) relative expression levels. [file Image_1.tif]
